# Identification of key genes and pathways associated with esophageal squamous cell carcinoma development based on weighted gene correlation network analysis

**DOI:** 10.7150/jca.30699

**Published:** 2020-01-13

**Authors:** Mingrui Shao, Wenya Li, Shiyang Wang, Zhenghua Liu

**Affiliations:** 1 Department of Thoracic Surgery, The First Affiliated Hospital of China Medical University, Shenyang, Liaoning Province, China.; 2 Department of Geriatric Surgery, The First Affiliated Hospital of China Medical University, Shenyang, Liaoning Province, China.

**Keywords:** esophageal squamous cell carcinoma, weighted gene correlation network analysis, risk, prognosis.

## Abstract

**Background**: As one of the most aggressive malignancies, esophageal squamous cell carcinoma(ESCC) remains one of the leading causes of cancer related death worldwide. The majority of ESCCs are diagnosed at advanced stages with poor five-year survival rate, making it urgent to identify specific genes and pathways associated with its initiation and prognosis.

**Materials and Methods**: The differentially expressed genes in TCGA were analysed to construct a co-expression network by WGCNA. Gene ontology (GO) terms and Kyoto Encyclopedia of Genes and Genomes (KEGG) pathways analysis were performed for the selected genes. Module-clinical trait relationships were analyzed to explore the genes and pathways that associated with clinicopathological parameters of ESCC. Log-rank tests and COX regression were used to identify the prognosis-related genes.

**Results**: The brown module containing 716 genes which most significantly contributed to ESCC. GO analysis suggested enrichment of adaptive immune response, cyclin-dependent protein serine, regeneration and mRNA metabolic process. KEGG analysis indicated pathways including Cellular senescence, Ribosome biogenesis, Proteasome, Base excision repair and p53 signaling pathway. Clinical stage was associated with cyan module; clinical M was associated with grey60 module; clinical T was associated with darkturquoise module; while clinical N, histological type and cancer location were associated with turquoise module. Key genes of TCP1, COQ3, PTMA and MAPRE1 might be potential prognostic markers for ESCC.

**Discussion**: Differentially expressed genes and key modules contributing to initiation and progression in ESCC were identified by WGCNA. These findings provide novel insights into the mechanisms underlying the initiation, prognosis and treatment of ESCC.

## Introduction

As one of the most aggressive malignancies, esophageal cancer remains one of the leading causes of cancer related death worldwide 1, 2. Among the two main histological types of esophageal cancer including esophageal squamous cell carcinoma (ESCC) and esophageal adenocarcinoma (EAC), ESCC represents the dominant subtype in Asians and EAC incidence is higher in Western countries 3. These two subtypes have different pathogenesis, biology features and clinical outcomes 4. At present, the majority of ESCCs are diagnosed at advanced stages, the five-year survival rate of which still remains unfavourable with frequently metastasis and recurrence 5, 6. Therefore, comprehensive researches are urgently needed to fully clarify critical molecular mechanisms related to the initiation, progression and prognosis of ESCC.

It has been widely accepted that alcohol drinking and tobacco consumption are certain risk factors for ESCC with synergistic effects 7, 8. There is also evidence suggesting that possible association of human papillomavirus (HPV) with ESCC risk according to meta-analysis 9. Studies have found that polymorphisms in aldehyde dehydrogenase 2 (ALDH2) gene also lead to the development of ESCC especially in Asian populations 10-12. Multiple studies including high-throughput analysis revealed that genes and pathways involved in ESCC were related with cell cycle, differentiation, and Epidermal Growth Factor Receptor signalling 13. In addition, epigenetic alterations including DNA methylation of such as APC, RB1 and CDKN2A, histone modification, and loss of genome imprinting also contribute to ESCC 14-16.

Although a number of genes and mechanisms have been proved to be closely implicated in the development of ESCC, the comprehensive picture of the whole genes and regulations of ESCC is still unclear. In recent years, bioinformatic methods become increasingly effective in exploration and analysis of multiple genes or proteins of complicated diseases. Weighted gene co-expression network analysis (WGCNA), a new gene co-expression network-based method, has been successfully used to screen biomarkers and pathways that could be applied in susceptibility genes, diagnose and treatment of cancer 17. In this study, WGCNA was conducted to analyse data of TCGA data repository of ESCC to identify gene modules and biomarkers (hub genes) implicated in the pathogenesis, progression and prognosis of ESCC.

## Materials and Methods

### Acquiring and preparing genetic and clinical data

The RNA sequencing and clinical data of esophageal squamous cell carcinoma patients were downloaded from TCGA data repository (https://cancergenome.nih.gov/). The level of gene expression was measured as fragments per kilobase of transcript per million mapped reads (FPKM). Clinical data included the sample type, clinical TNM stage, histologic grade, cancer position and survival information. Samples with incomplete pathologic stage or histologic grade information were not included. As genes with little variation in expression usually represent noise, the most variant genes were filtered for network construction. Gene variabilities were measured by median absolute deviation (MAD).

### Constructing gene co-expression network

Gene co-expression network was constructed by the WGCNA package in R and was visualized by Cytoscape software 18. Power values were screened out by WGCNA algorithm in the construction of co-expression modules. Scale independence and average connectivity analysis of modules with different power value were performed by gradient test (power value ranging from 1 to 20). Appropriate power value was determined when the scale independence value was equal to 0.9. WGCNA algorithm was then used to construct the co-expression network and extract the gene information in the most relevant module. The criterion of co-expression weight> 2 was used to select the candidate network. Heatmap tool package in R language was adopted to describe the strength of the relationship among modules (strong or weak degree).

### Relating modules to cancer risk and identifying the prognosis related genes

One of the advantages of WGCNA is that the correlation between modules and clinical parameters can be analyzed. The module eigengene (ME), which can be regarded as a representative of the gene expression profiles from a module, is defined as the first principal component of a given module. Given that the ME can summarize the gene expression profiles, we calculated the correlation between MEs and external sample type data. The gene in the most relevant module was chosen as the risk-related gene. Log-rank tests and COX regression were used to select the prognosis-related genes in the risk-related genes. R package survival was used to carry out log-rank tests and survminer package was used to plot Kaplan-Meier survival curves. In order to validate our findings, microarray dataset of GSE53625 from Gene Expression Omnibus (GEO, https://www.ncbi.nlm.nih.gov/geo/) in NCBI (The National Center for Biotechnology Information) containing 179 esophageal squamous cell carcinoma patients with prognosis information were used to confirm our results.

### Gene ontology and pathway Enrichment analysis

To explore the potential biological themes and pathways of genes in risk-related module, the clusterprofiler package in R was used to annotate and visualize gene ontology (GO) terms 19 and Kyoto Encyclopedia of Genes and Genomes (KEGG) pathways 20.

### Screening for candidate module with clinical significance and KEGG analysis

We calculated the correlation between MEs and external clinical data. *p <*0.05 suggested significant correlation. We selected the most relevant module in each clinical data for analysis. KEGG pathway analysis was used to enrich the related genes.

## Results

### Gene co-expression network of ESCC

**Figure 1:**
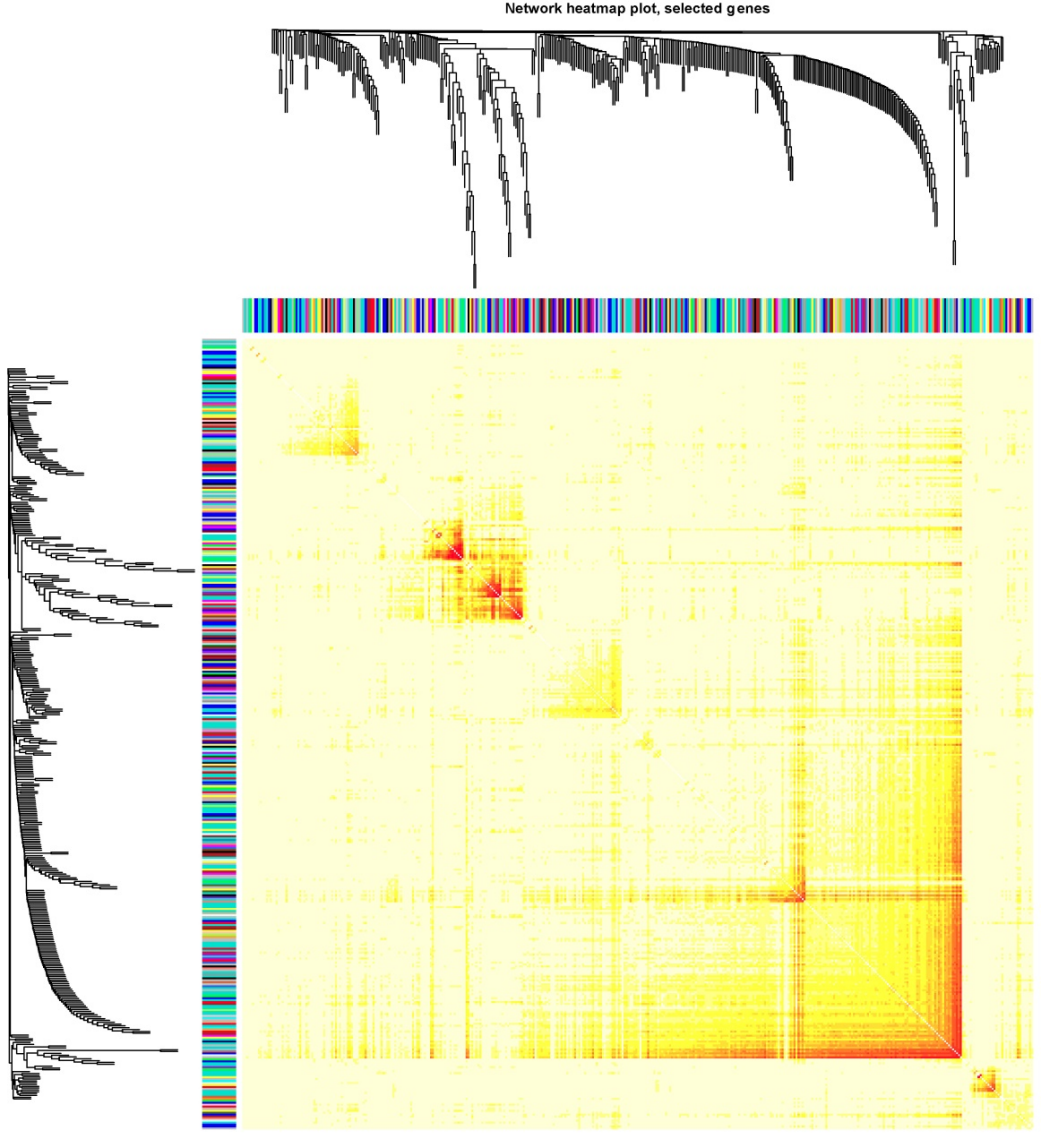
The connectivity analysis of critical genes in different module

Clinical and level-3 RNA sequencing data for 162 esophageal squamous cell carcinoma samples and 11 normal samples were obtained from TCGA database. For module detection, the 15121 most variant genes were selected according to MAD value for further analysis. When the value of soft thresholding power *β* was 4, the connectivity between genes met a scale-free network distribution (Fig. S1). Altogether 21 modules were identified by hierarchical clustering and the Dynamic branch Cutting. Each module was assigned a unique color as an identifier. Interaction relationship analysis of co-expression genes was shown in Figure 1. The number of genes in modules ranged from 34 to 2353. The grey module represented a gene set that was not assigned to any of the modules.

### The risk related module in the WGCNA algorithm

**Figure 2:**
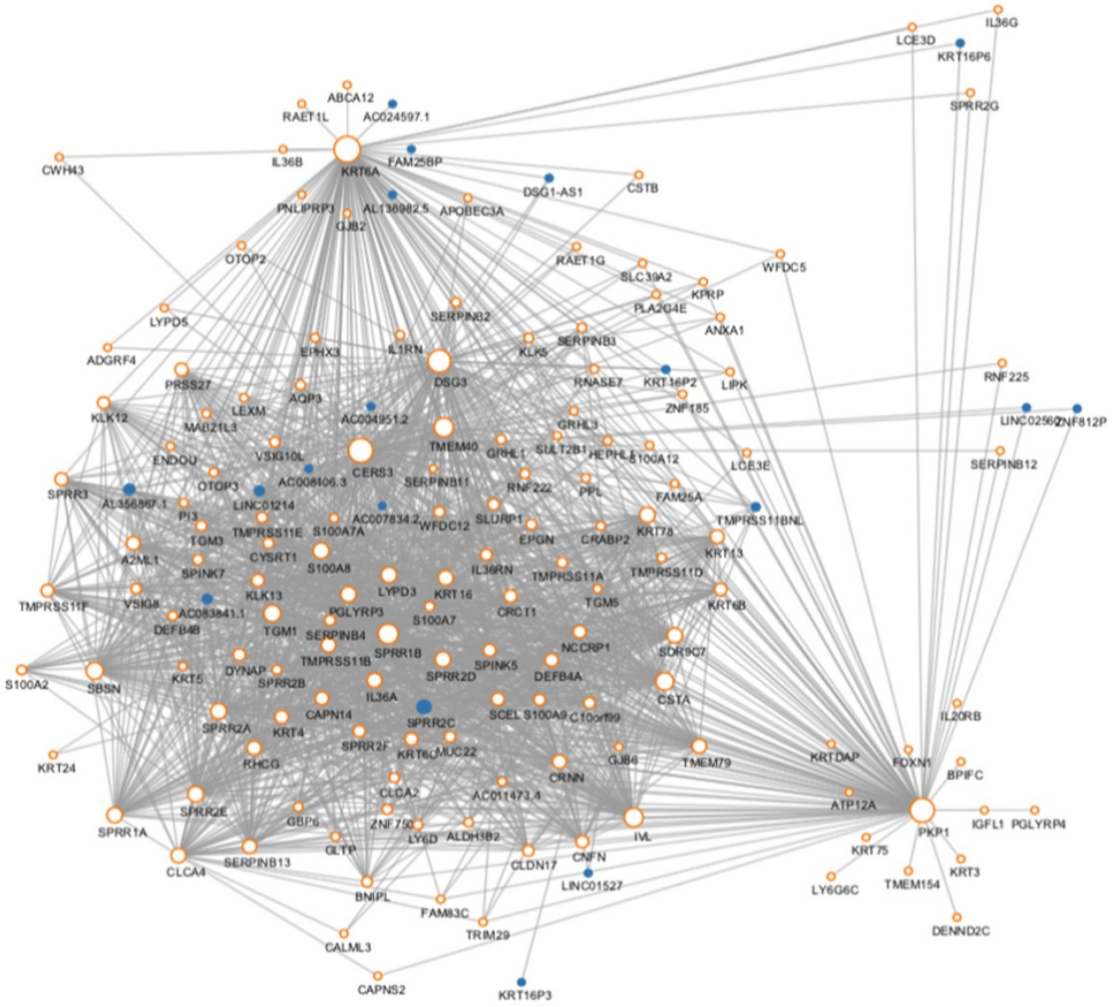
The protein-protein interaction network of the genes in the risk-related brown module of ESCC.

We then use the cancer type as the Clinical phenotype to select the most relevant module with the occurrence of ESCC. Finally, the brown module was selected (Fig. S2). The brown module contained 716 genes. Then we constructed the co-expression network with the criterion of co-expression weight >2. As was shown in Figure 2, a total of 161 genes were selected and their multiple interactions were visualized. The size represents the connection numbers and the color represents the fold change compared by the cancer and normal samples.

### Enrichment analysis of the brown module

**Figure 3:**
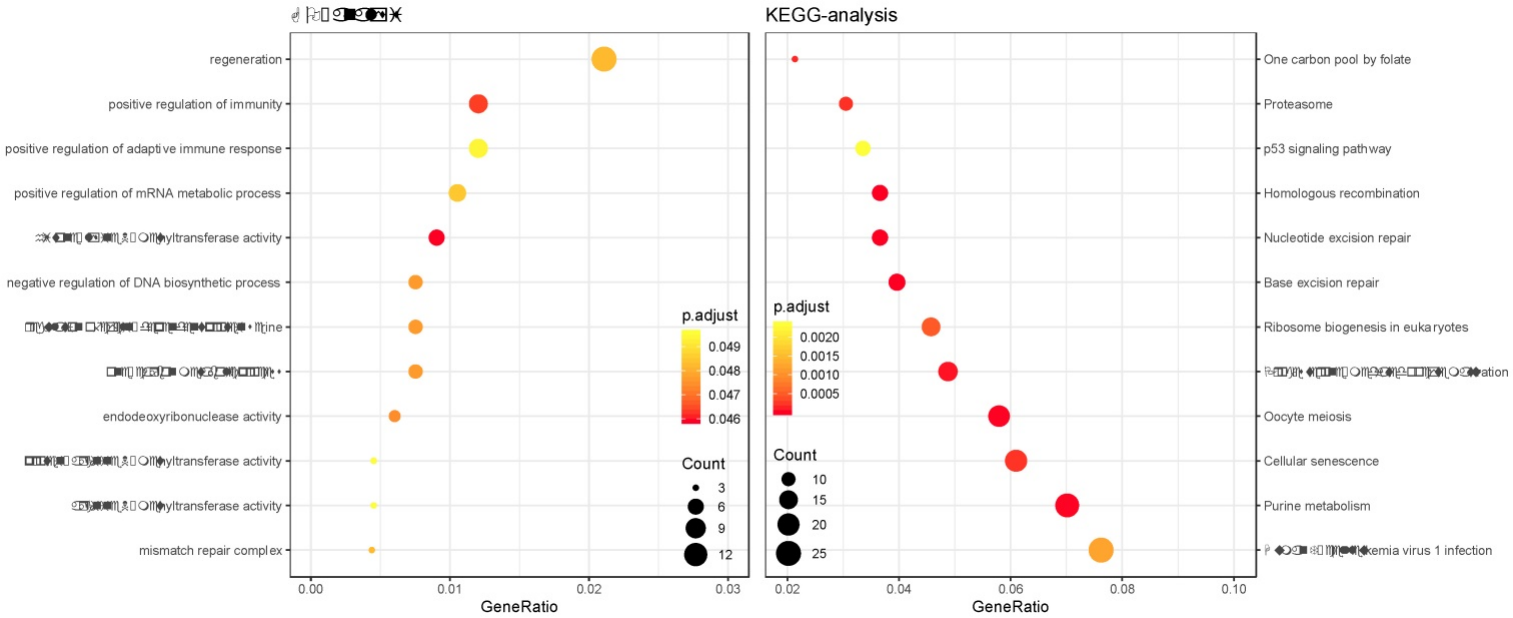
Gene Ontology analysis and KEGG pathway enrichment analysis for genes in the risk-related brown module of ESCC.

GO and KEGG enrichment analysis was performed on the genes in the constructed cancer-risk-related module. Altogether 984 terms showed difference in GO enrichment (Table 1). As was illustrated in Figure 3, this module in GO analysis was related with biological process of regulation of adaptive immune response, regulation of cyclin-dependent protein serine, regeneration and positive regulation of mRNA metabolic process. According to the KEGG analysis, 19 pathways were associated with brown module including Cellular senescence, Ribosome biogenesis in eukaryotes, Proteasome, Base excision repair and p53 signaling pathway.

### Prognosis analysis of the brown module

**Figure 4:**
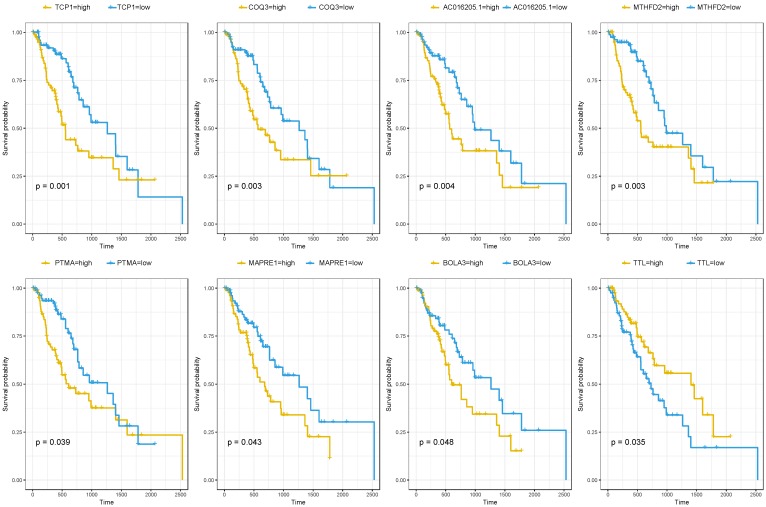
The correlation between the expression levels of key genes of risk-associated brown module and the survival of ESCC patients.

Since the brown module were selected from the modules related to patients' cancer type. It was of great significance to evaluate the potentiality of them to serve as prognostic biomarkers. Among the 716 genes, 39 genes were associated with the prognosis of esophageal squamous cell carcinoma (Table 2). As was summarized in Figure 4, the top four up-regulated genes which indicated a poor prognosis included TCP1 (HR=2.427, P=0.001), COQ3 (HR=2.247, P=0.003), AC016205.1 (HR=2.227, P=0.004) and MTHFD2 (HR=2.179, P=0.003). Of the top four down-regulated genes, PTMA (HR=1.706, P=0.039), MAPRE1 (HR=1.698, P=0.043) and BOLA3 (HR=1.667, P=0.048) significantly associated with poor prognosis while TTL predicted better survival (HR=0.572, P=0.035).

Altogether 179 esophageal squamous cell carcinoma patients with prognosis information of microarray GSE53625 were used to validate our results. As was shown in Table 4, AC016205.1 (P=0.013), PTMA (P=0.023), TTL (P=0.013) still showed significant relation with prognosis of ESCC patients, while MTHFD2 (P=0.082) indicated borderline significant association (Table 4).

### Module-clinical trait relationships

**Figure 5:**
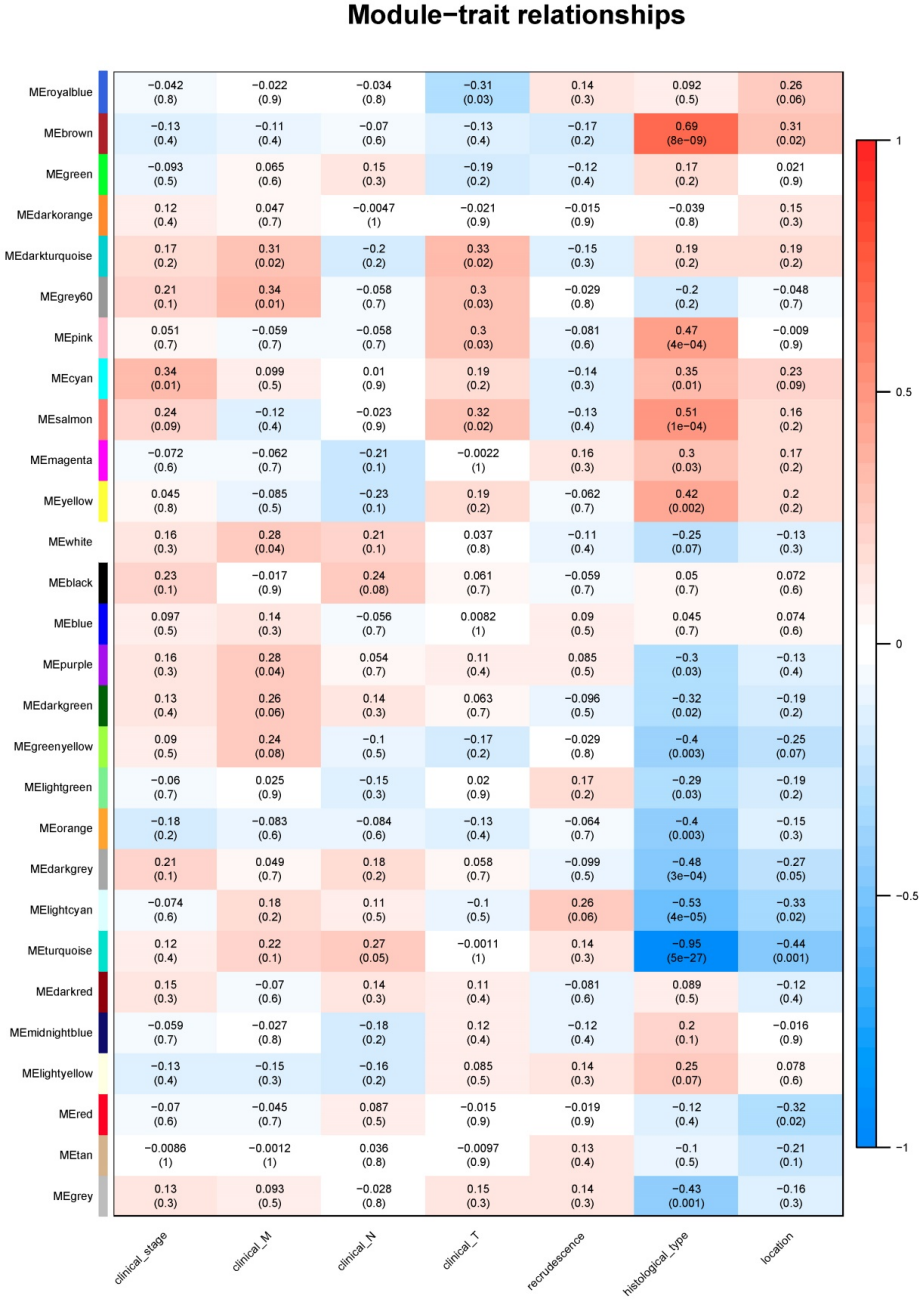
The module-clinical trait relationships of genes involved in ESCC.

Identifying genes associated with a certain clinical trait is of great value to explore the molecular mechanisms behind the trait. In the present study, the clinical parameters of ESCC patients, including clinical T, clinical N, clinical M, clinical stage, recrudescence stage, histological type and cancer location were involved in the module-trait relationship (MTR) analysis. As was suggested in Figure 5, clinical stage was associated with cyan module; clinical M was associated with grey60 module; clinical T was associated with darkturquoise module; while clinical N, histological type and cancer location were associated with turquoise module. No module correlated with recrudescence.

### KEGG analysis of clinical related module

We then performed KEGG pathway analysis of the candidate module. The results indicated that genes of cyan module were enriched in Cell cycle; grey60 module was associated with Ribosome; darkturquoise module was related with RNA transport; turquoise module was associated with Fat digestion and absorption, Tight junction, Maturity onset diabetes of the young, Protein digestion and absorption and Fructose and mannose metabolism (Table 3).

## Discussion

Although significant progresses have been made in studies concerning the risk and development of ESCC, our understanding of the complex mechanisms of ESCC is still limited. In this study, we conducted a differential expression analysis followed by WGCNA to detect genes and pathways associated with the occurrence, clinical parameters and prognosis of ESCC. Finally, key modules were identified which contribute to the risk (brown module) and clinicopathological parameters (cyan module, grey60 module, darkturquoisemodule and turquoise module) of ESCC. In addition, enrichment analysis of the genes in core modules suggested significant involvement of pathways such as Cellular senescence, Ribosome biogenesis in eukaryotes, Proteasome, Base excision repair and p53 signalling pathway.

In this present study, RNA sequencing data for 162 esophageal squamous cell carcinoma samples and 11 normal samples from TCGA were systematically analyzed. According to the WGCNA analysis of most variant genes, altogether 21 modules were identified and each module was assigned a unique color as an identifier. The number of genes in modules ranged from 34 to 2353. The brown module containing 716 genes was selected when we used the cancer type as the Clinical phenotype, indicating its close involvement in the development of ESCC. GO enrichment analysis were then performed on the genes in the constructed cancer-risk-related module, suggesting significance of biological process including adaptive immune, cyclin-dependent protein serine, regeneration and mRNA metabolic process. It has been reported that immune response and immune escape might play a critical role in ESCC progression and therapy 21, 22. In addition, key mRNA metabolic procedure such as alternative splicing has also been detected in ESCC. Alternative splicing isoforms of LOXL2, VIL2, OSMRβ and MUC1 have been reported to contribute to ESCC development and progression 23-26. From this point of view, the specific role of immune response and altered mRNA metabolic process in ESCC require future investigations to elucidate. As for key pathways of KEGG enrichment, aberrant base excision repair capacity and altered p53 signaling pathways have been found to be associated with ESCC development by a number of studies 27, 28. Pathways of cellular senescence, ribosome biogenesis in eukaryotes and proteasome might be novel research directions for physicians and surgeons for ESCC.

The five-year survival rate of ESCC currently remains unsatisfactory despite recent improvements in treatments of surgical resection and adjuvant chemotherapy. It was therefore of great significance to evaluate the potentiality of key factors to serve as prognostic biomarkers. We eventually screened 39 prognosis-related genes in ESCC after analyzing the risk-associated brown module. Aberrant TCP1 gene status has been detected in hepatocellular carcinoma, breast cancer and colorectal cancer, but no study has been performed for its effect in ESCC 29-31. MTHFD2 RNA and protein are remarkably increased in many types of cancers and correlated with worse survival in breast cancer according to a comprehensive analysis of RNA profiles of 1,454 metabolic enzymes across 1,981 tumors spanning 19 cancer types 32. Although some of the prognosis-related genes we identified have been investigated in various types of cancers, limited information was known about their roles in ESCC until now. One limitation of this study is that most results were generated by analysis of publicly available data, further studies based on larger populations and molecular mechanism researches are therefore needed to confirm the results of our study.

In order to explore the genes and modules that associated with clinicopathological parameters of ESCC, we also performed module-clinical trait relationships analysis. We finally detected specific modules which determine the clinical stage, TNM classification, histological type and cancer location. Pathway analysis of the selected modules indicated that these genes enriched in Cell cycle, Ribosome, RNA transport, Fat digestion and absorption, Tight junction, Maturity onset diabetes of the young, Protein digestion and absorption and Fructose and mannose metabolism. Remodeling of energetic metabolism is a hallmark of malignant tumor, by which tumor cells changes themselves and the environment into more suitable conditions for growth and invasion 33. According to our findings, aberrant metabolism of carbohydrate, fat and protein might be closely related to the progression of ESCC. In addition, alternations in functions of ribosome, RNA transport might also be promising research directions for ESCC.

In summary, differentially expressed genes and key modules contributing to initiation and progression in ESCC were identified by means of WGCNA. Pathways including cellular senescence, ribosome biogenesis in eukaryotes, Proteasome, base excision repair, fat digestion and absorption and p53 signalling might be closely related with ESCC development. Key genes of TCP1, COQ3, PTMA and MAPRE1 might be potential prognostic markers for ESCC. These findings provide novel insights into the mechanisms underlying the etiology, prognosis and treatment of ESCC.

## Supplementary Material

Supplementary figures and tables.

### Acknowledgements

This study was supported by Natural Science Foundation of Liaoning Province (grant no. 2015020561), Wu JiePing Medical Found (grant no. 320.6750.18293) and the Fund for Scientific Research of The First Hospital of China Medical University (grant no. fsfh1514).

## Figures and Tables

**Table 1:** Top 5 enrichment analysis of the brown module

Ontology	ID	Description	GeneRatio	adjusted P	Count
BP	GO:0002821	positive regulation of adaptive immune response	8/664	0.049372	8
BP	GO:1903313	positive regulation of mRNA metabolic process	7/664	0.0485	7
BP	GO:0031099	regeneration	14/664	0.048208	14
BP	GO:0006730	one-carbon metabolic process	5/664	0.047667	5
BP	GO:0045737	positive regulation of cyclin-dependent protein serine/threonine kinase activity	5/664	0.047667	5
CC	GO:0030687	preribosome, large subunit precursor	4/684	0.037033	4
CC	GO:0000932	P-body	8/684	0.035763	8
CC	GO:0000315	organellar large ribosomal subunit	6/684	0.035241	6
CC	GO:0005762	mitochondrial large ribosomal subunit	6/684	0.035241	6
CC	GO:0036464	cytoplasmic ribonucleoprotein granule	14/684	0.033517	14
MF	GO:0016273	arginine N-methyltransferase activity	3/663	0.049595	3
MF	GO:0016274	protein-arginine N-methyltransferase activity	3/663	0.049595	3
MF	GO:0016889	endodeoxyribonuclease activity, producing 3'-phosphomonoesters	4/663	0.047419	4
MF	GO:0018024	histone-lysine N-methyltransferase activity	6/663	0.045899	6
MF	GO:0004540	ribonuclease activity	10/663	0.045899	10
KEGG	hsa04115	p53 signaling pathway	11/328	0.002355	11
KEGG	hsa05166	Human T-cell leukemia virus 1 infection	25/328	0.001213	25
KEGG	hsa03008	Ribosome biogenesis in eukaryotes	15/328	0.000475	15
KEGG	hsa04218	Cellular senescence	20/328	0.000221	20
KEGG	hsa03050	Proteasome	10/328	0.000207	10

**Table 2:** Prognosis analysis of the brown module

Gene	Gene full name	Location	HR	Lower limit	Upper limit	P
TCP1	t-complex 1	6q25.3-q26	2.427	1.439	4.098	0.001
MTHFD2	methylenetetrahydrofolate dehydrogenase (NADP+ dependent) 2	2p13.1	2.179	1.302	3.650	0.003
COQ3	coenzyme Q3, methyltransferase	6q16.2	2.247	1.318	3.831	0.003
AC016205.1	MIR924 Host Gene	18q12.2	2.227	1.284	3.861	0.004
VBP1	VHL binding protein 1	Xq28	2.088	1.230	3.546	0.006
MED30	Mediator Complex Subunit 30	8q24.11	2.110	1.208	3.690	0.009
SNRPC	small nuclear ribonucleoprotein polypeptide C	6p21.31	1.976	1.178	3.311	0.010
NONO	non-POU domain containing, octamer-binding	Xq13.1	1.972	1.170	3.333	0.011
SNRPB	small nuclear ribonucleoprotein polypeptides B and B1	20p13	1.961	1.157	3.311	0.012
NUDT21	nudix hydrolase 21	16q12.2	1.992	1.164	3.413	0.012
ALYREF	Aly/REF Export Factor	17q25.3	1.887	1.138	3.125	0.014
CCT4	chaperonin containing TCP1 subunit 4	2p15	1.931	1.133	3.289	0.016
IRAK1	interleukin 1 receptor associated kinase 1	Xq28	1.866	1.110	3.135	0.019
AHSA1	activator of Hsp90 ATPase activity 1	14q24	1.832	1.095	3.058	0.021
CDT1	chromatin licensing and DNA replication factor 1	16q24.3	1.835	1.095	3.067	0.021
CCT2	chaperonin containing TCP1 subunit 2	12q15	1.789	1.080	2.967	0.024
HNRNPD	heterogeneous nuclear ribonucleoprotein D	4q21	1.808	1.082	3.021	0.024
SAAL1	Serum Amyloid A Like 1	11p15.1	1.783	1.073	2.959	0.026
SNRPE	small nuclear ribonucleoprotein polypeptide E	1q32	1.805	1.074	3.040	0.026
SPDL1	spindle apparatus coiled-coil protein 1	5q35.1	1.789	1.066	3.003	0.028
YARS2	tyrosyl-tRNA synthetase 2	12p11.21	1.795	1.063	3.030	0.029
E2F7	E2F Transcription Factor 7	12q21.2	1.828	1.055	3.165	0.031
MRPL18	mitochondrial ribosomal protein L18	6q25.3	1.733	1.048	2.865	0.032
LSM5	LSM5 homolog, U6 small nuclear RNA and mRNA degradation associated	7p14.3	1.745	1.049	2.907	0.032
TTL	Tubulin Tyrosine Ligase	2q14.1	0.572	0.340	0.962	0.035
PTGES3	prostaglandin E synthase 3	12q13.3	1.733	1.040	2.890	0.035
SRSF2	serine and arginine rich splicing factor 2	17q25.1	1.718	1.040	2.841	0.035
HSPD1	heat shock protein family D (Hsp60) member 1	2q33.1	1.718	1.031	2.865	0.038
SF3A3	splicing factor 3a subunit 3	1p34.3	1.712	1.029	2.849	0.039
PTMA	prothymosin, alpha	2q37.1	1.706	1.026	2.833	0.039
CTPS1	CTP synthase 1	1p34.1	1.684	1.022	2.770	0.041
MAGOHB	mago homolog B, exon junction complex core component	12p13.2	1.709	1.020	2.865	0.042
MAPRE1	microtubule associated protein RP/EB family member 1	20q11.1-q11.23	1.698	1.016	2.833	0.043
MCM8	minichromosome maintenance 8 homologous recombination repair factor	20p12.3	1.692	1.014	2.825	0.044
TRIM37	tripartite motif containing 37	17q23.2	0.596	0.360	0.989	0.045
HNRNPAB	heterogeneous nuclear ribonucleoprotein A/B	5q35.3	1.669	1.010	2.762	0.046
STRAP	serine/threonine kinase receptor associated protein	12p12.3	1.706	1.004	2.907	0.048
BOLA3	BolA Family Member 3	2p13.1	1.667	1.005	2.762	0.048
LSM6	LSM6 homolog, U6 small nuclear RNA and mRNA degradation associated	4q31.22	1.698	1.005	2.865	0.048

**Table 3:** KEGG analysis of clinical related module

Phenotype	Module	ID	Description	GeneRatio	Adjusted P	Count
Clinical stage	cyan	hsa04110	Cell cycle	4/29	0.0012644	4
Clinical M	grey60	hsa03010	Ribosome	4/24	0.0013257	4
Clinical T	darkturquoise	hsa03013	RNA transport	6/45	0.0005229	6
Clinical NHistological typeCancer location	turquoise	hsa04975	Fat digestion and absorption	14/607	2.21E-06	14
		hsa04530	Tight junction	31/607	1.499E-05	31
		hsa04950	Maturity onset diabetes of the young	10/607	1.936E-05	10
		hsa04974	Protein digestion and absorption	20/607	2.754E-05	20
		hsa00051	Fructose and mannose metabolism	11/607	3.566E-05	11

**Table 4:** Verification for top 4 prognosis gene in GEO datasets

Gene	Gene full name	HR(95% CI)	P	GSE53625_P
TCP1	t-complex 1	2.427(1.439-4.098)	0.001	0.196
MTHFD2	methylenetetrahydrofolate dehydrogenase (NADP+ dependent) 2	2.179(1.302-3.65)	0.003	0.082
COQ3	coenzyme Q3, methyltransferase	2.247(1.318-3.831)	0.003	0.130
AC016205.1	MIR924 Host Gene	2.227(1.284-3.861)	0.004	**0.013**
PTMA	prothymosin, alpha	1.706(1.026-2.833)	0.039	**0.023**
MAPRE1	microtubule associated protein RP/EB family member 1	1.698(1.016-2.833)	0.043	0.519
BOLA3	BolA Family Member 3	1.667(1.005-2.762)	0.048	0.781
TTL	Tubulin Tyrosine Ligase	0.572(0.34-0.962)	0.035	**0.013**
