# Supplementary material for: Identification of key genes and pathways associated with esophageal squamous cell carcinoma development based on weighted gene correlation network analysis
Source: J Cancer. 2020 Jan 13;11(6):1393–402. doi: 10.7150/jca.30699 (PMC6995384; doi:10.7150/jca.30699)

Supplemetary Figure 1. Soft Threshold Selection for WGCNA Analysis

Supplemetary Figure 1

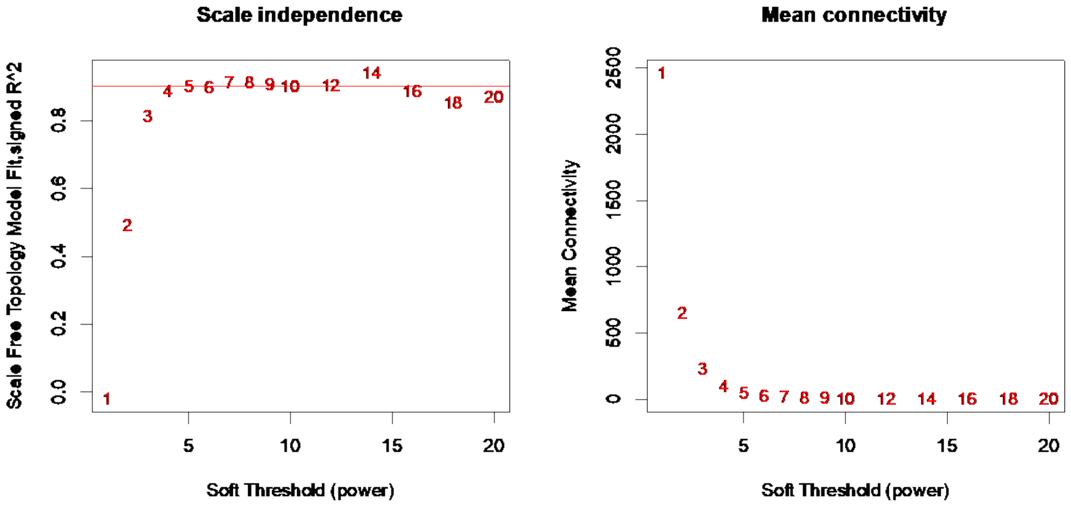

Supplemetary Figure 2. The module–clinical trait relationships of genes involved in cancer risk related ESCC.

Supplemetary Figure 2

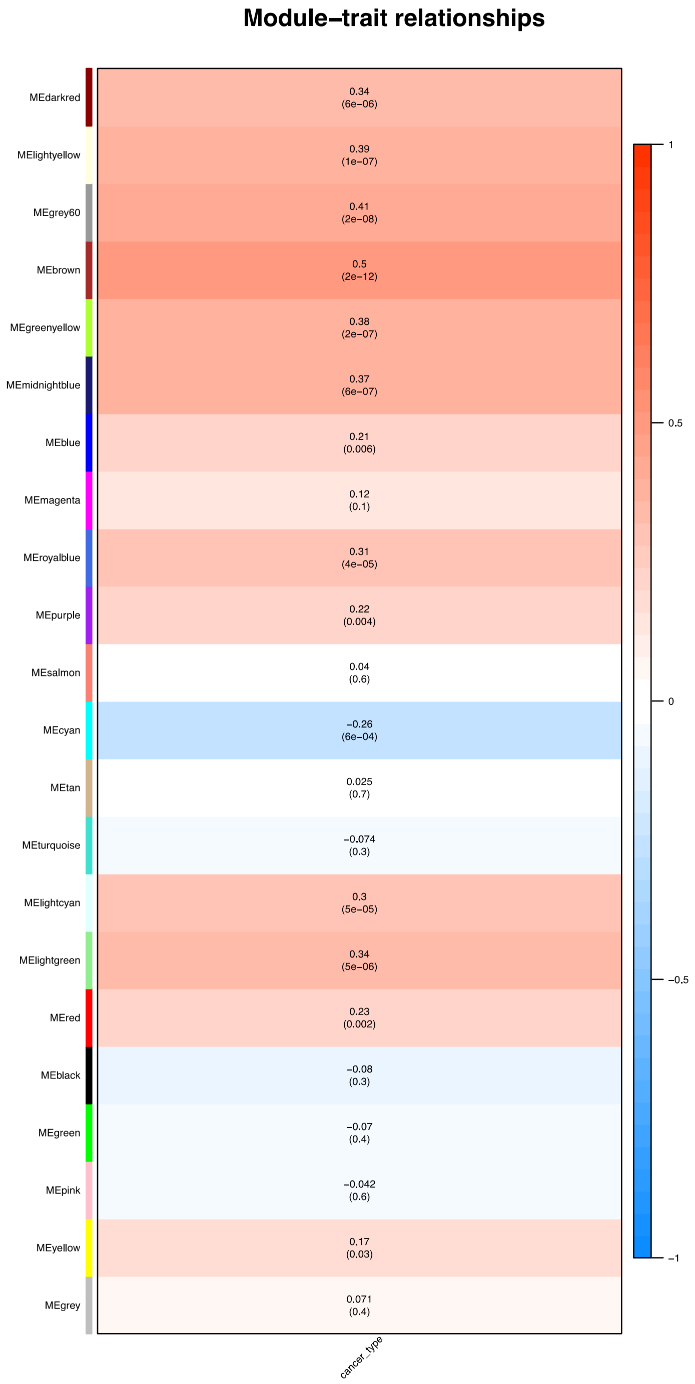

Supplement: Supplementary file 1 — Supplementary figures and tables. [file jcav11p1393s1.pdf]
